# Smooth Pursuit Eye Movements Improve Temporal Resolution for Color Perception

**DOI:** 10.1371/journal.pone.0011214

**Published:** 2010-06-21

**Authors:** Masahiko Terao, Junji Watanabe, Akihiro Yagi, Shin'ya Nishida

**Affiliations:** 1 NTT Communication Science Laboratories, NTT Corporation, Kyoto, Japan; 2 Department of Life Sciences, University of Tokyo, Meguro-ku, Tokyo, Japan; 3 Graduate School of Humanities, Kwansei Gakuin University, Nishinomiya, Japan; 4 Japan Society for the Promotion of Science, Chiyoda-ku, Tokyo, Japan; University of Minnesota, United States of America

## Abstract

Human observers see a single mixed color (yellow) when different colors (red and green) rapidly alternate. Accumulating evidence suggests that the critical temporal frequency beyond which chromatic fusion occurs does not simply reflect the temporal limit of peripheral encoding. However, it remains poorly understood how the central processing controls the fusion frequency. Here we show that the fusion frequency can be elevated by extra-retinal signals during smooth pursuit. This eye movement can keep the image of a moving target in the fovea, but it also introduces a backward retinal sweep of the stationary background pattern. We found that the fusion frequency was higher when retinal color changes were generated by pursuit-induced background motions than when the same retinal color changes were generated by object motions during eye fixation. This temporal improvement cannot be ascribed to a general increase in contrast gain of specific neural mechanisms during pursuit, since the improvement was not observed with a pattern flickering without changing position on the retina or with a pattern moving in the direction opposite to the background motion during pursuit. Our findings indicate that chromatic fusion is controlled by a cortical mechanism that suppresses motion blur. A plausible mechanism is that eye-movement signals change spatiotemporal trajectories along which color signals are integrated so as to reduce chromatic integration at the same locations (i.e., along stationary trajectories) on the retina that normally causes retinal blur during fixation.

## Introduction

Alternation of two colors appears to be fused into a single mixed color when the alternation rate exceeds so-called critical chromatic flicker frequency. It is typically around 15 Hz, though being significantly variable by stimulus condition [1], [2]. Exploiting this temporal limitation of human color perception, recent digital displays, such as DLP projectors and plasma displays, show millions of colors by rapidly modulating tri-color light sources. Although chromatic fusion has been known for centuries, the neural mechanisms that determine the fusion frequency remain poorly understood. A classical view ascribes the fusion frequency to the temporal resolution of color encoding by peripheral retinal sensors, but accumulating evidence suggests that temporal changes beyond the fusion frequency can activate cortical neurons [3]–[7]. Although these finding indicate that the chromatic fusion is at least to some extent a cortical phenomenon, the involved cortical processes remain unspecified.

Here we report a psychophysical finding that indicates a cortical process for color fusion control. We found that while human observers were making a smooth pursuit eye movement to track a target moving on a stationary background, they showed improved fusion frequencies in detection of the background pattern. This finding suggests that the chromatic critical fusion frequency can be elevated by a cortical process related to visibility improvement during eve movements.

The pursuit eye movement can keep a tracked object in the fovea, but it also introduces a backward retinal sweep of stationary background patterns. The motion of a fine-textured background produces a rapid stimulus change in the retinal image, and low temporal resolution of the color mechanism would introduce severe motion blur. However, since the brain knows how it moves the eyes and how the stationary background would move on the retina, it is theoretically possible for the brain to overcome the motion blur [8] more effectively than in other cases. Our finding indicates that the brain in fact does this excellent job not only by reducing subjective blur [9], but also by objectively improving chromatic temporal resolution. In addition, the effects of pursuit eye movements we observed cannot be explained by the general gain increase of parvocellular mechanisms suggested recently [10].

## Results

**Figure 1:**
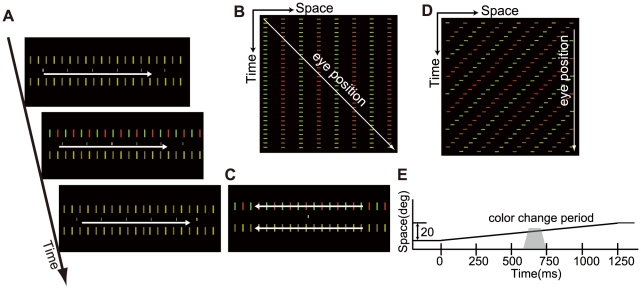
Experiment to test the effect of pursuit on the chromatic fusion frequency. (A) Time course of a stimulus of the pursuit condition. A chromatic target is presented in the upper array at the middle of the sequence. A white arrow indicates the movement of the pursuit target. (B) Space-time plot the pursuit condition for the period when the chromatic target is presented. The horizontal axis is the horizontal position on the display. On the retina, the space-time plot becomes identical to (E). (C) Schematic illustration of temporal sequence of stimuli. A chromatic target appeared at the middle of a stimulus presentation, when the tracking point reached the screen center under the pursuit condition, which made the target retinal pattern similar for the two conditions. (D) Spatial configuration of the fixation condition. White arrows indicate the movement of bars. (E) Space-time plot of a chromatic target of the fixation condition. The horizontal axis is the horizontal position on the display and on the retina. The gray shaded area indicates a color change period.

We presented on a dark background two arrays of bright bars, one above and the other below the fixation point. The bar arrays were yellow except when a chromatic target was presented. Under the pursuit condition (Figure 1A, B), the arrays were always stationary on the display. The observer tracked a fixation point that smoothly moved from the left end to the right end of the screen. When the fixation point reached the screen center area, either the upper or lower array briefly changed into a red-green grating at one of two levels of chromatic contrast (Figure 1C). During the target presentation, the observer's eye movement generated an alternation of red and green on the retina, at a rate varying between 13.3 to 26.7 Hz depending on the inter-bar spacing. The observers had to judge in which array the color change occurred. Under the fixation condition (Figure 1 D, E), the observers viewed moving bar arrays while fixating on the central stationary point and detected a similar color change. The two conditions produced nearly the same retinal image at the time of target presentation (Figure 1 E). Using the method of constant stimuli, we estimated the critical chromatic fusion frequency from the 75% correct point of the best fitting logistic function.

**Figure 2:**
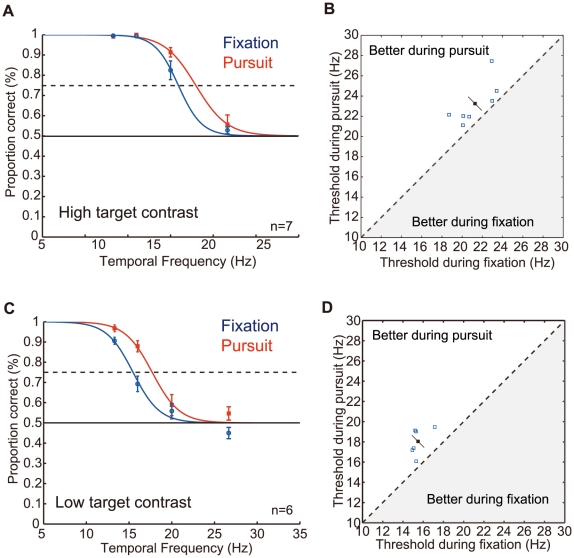
The critical chromatic fusion frequencies obtained under the pursuit condition and the fixation condition. (A and B) The results obtained with the full chromatic contrast target. (C and D) The results obtained with the half chromatic contrast target. (A and C) Psychometric function for the data averaged across observers. Dashed line indicates the 75% correct line that specifies the critical fusion frequency. (B and D) Scatter plot of the individual thresholds (blue open squares), and their mean (a black filled square) with 95% confidential interval. The data above the diagonal line indicates that the critical fusion frequency obtained during pursuit is better than that obtained during fixation. All the data are in this range.

The results (Figure 2) indicate a small but robust improvement of chromatic target detection while the observer was making pursuit. The psychometric function of the pursuit condition, computed from the responses of the all the observers, was shifted to the higher temporal frequency relative to that the fixation condition. The shift was ∼2 Hz for both high and low target contrast conditions (Figure 2A and C, respectively). Scatter plots of individual detection thresholds (Figure 2B and D) indicate that the critical fusion frequency was always higher during pursuit than during fixation. The difference in the individual average was statistically significant: mean ±1 standard error was 23.2±0.8 Hz vs. 21.3±0.7 Hz for full chromatic-contrast targets, and 18.0±0.6 Hz vs. 15.5±0.3 Hz for half contrast targets (*p*<0.001 in both cases; two-tailed paired t-test).

The difference of retinal image between fixation and pursuit might cause the improvement of chromatic target detection. To minimize the difference in the magnitude of retinal jitter between the two stimulus conditions, we computed the psychometric function of the pursuit condition only from those trials in which retinal deviation from the expected pursuit trajectory was within the range shown by the jitter distribution of the fixation condition. Although equating the jitter range did not equate the mean magnitude of jitters, even when we equated the shape of jitter distribution by re-sampling the data, the results remained essentially the same. Even with a small jitter range, if the pursuit were consistently slower than the pursuit target (i.e., pursuit gain <1.0), the actual temporal frequency of the display could be lower than intended. However, for trials used for computing psychometric functions, the individual average (±1 standard deviation) of the pursuit gain during the target presentation period was 1.002 (±0.023) for high-contrast target, and 1.002 (±0.028) for low-contrast target. In addition, there was no significant correlation between the pursuit gain of each observer and his/her magnitude of improvement in temporal resolution (*r* = −0.14, *p* = 0.69).

**Figure 3:**
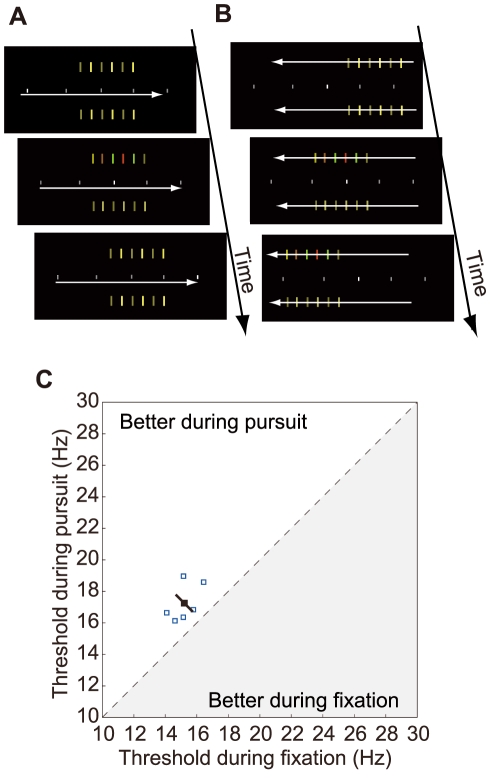
Experiment in which retinal image was equated for the whole stimulus sequence. (A) Spatial configuration of the display for the fixation condition. The chromatic target had the full contrast at the center. (B) Spatial configuration of the display for the pursuit condition. (C) Scatter plot of individual thresholds, which indicates higher critical fusion frequency during pursuit than during fixation, as in Figure 2.

In the first experiment, we presented stimulus arrays using the full width of the display. The retinal image was almost the same between the pursuit and fixation conditions at the middle of a stimulus sequence, but not exactly so before and after the target presentation since both ends of the arrays were always fixed on the display screen. To clear a concern that this minor stimulus difference might produce detection improvement for the pursuit condition, the second experiment equated the whole retinal image sequence including the pre- and post-target periods (Figure 3 A B). The results (Figure 3 C) again indicate a robust improvement in chromatic temporal resolution for the pursuit condition relative to the fixation condition (17.3±0.5 Hz vs. 15.2±0.3 Hz, p<0.001, two-tailed paired t-test).

A previous study from our group [11] showed that the chromatic fusion frequency is higher when measured with a motion stimulus (which was similar to that used for the current fixation condition) than when measured with flicker stimuli. That study ascribed this improvement to the operation of a hypothetical neural mechanism that suppresses motion blur [12] by way of integrating color signals along motion trajectory [13]. The first two experiments of the present study show that the chromatic fusion frequency is further elevated when the retinal motion is produced by the observer's pursuit. This finding is in line with a previous report that showed the magnitude of subjective motion blur is reduced during pursuit [9]. The present finding can be interpreted to imply that pursuit improves chromatic temporal resolution of the background pattern that is stationary in the environment, possibly through interaction with the trajectory integration mechanism.

**Figure 4:**
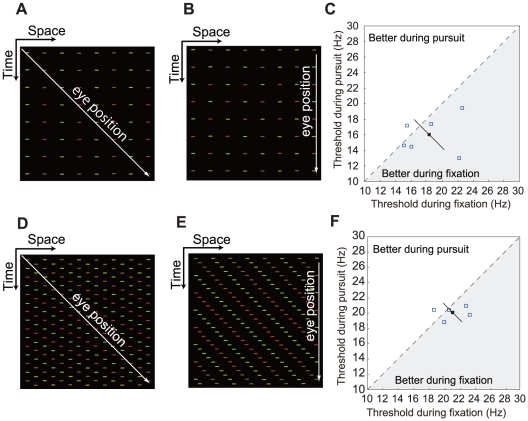
The temporal improvement is not a result of the general increase in chromatic contrast sensitivity. (A–C) The temporal improvement is not observed with non-directional flicker. (A) Space–time plot of the pursuit condition. (B) Space-time plot of the fixation conditions. (C) Scatter plot of individual thresholds, which indicates that critical fusion frequency was not higher during pursuit than during fixation. (D–F) The temporal improvement is direction selective. (D) Spatiotemporal configuration of the display in which the bars move twice as fast as the pursuit target. (E) Space–time plot of the retinal image was identical to that produced by stationary bars except that the direction was identical, rather than opposite, to the pursuit. (F) Scatter plot of individual thresholds, which indicates that critical fusion frequency was not higher during pursuit than during fixation.

However, an alternative interpretation of the present effect is suggested by a recent study by Schütz et al. [10], [14]. They found chromatic contrast sensitivity is enhanced during pursuit and ascribed this sensitivity change to an increased contrast gain of parvocellular mechanisms. It is possible that this increased contrast gain elevates chromatic critical flicker frequency, since the sensitivity enhancement was observed even for a 16.6 Hz flicker [10], and we also observed an increase in fusion frequency when the physical chromatic contrast was raised. In the study by Schütz et al. [10], [14], the contrast sensitivity change was observed with non-moving stimuli (flashed horizontal lines). If the general contrast sensitivity enhancement were the main mechanism of increasing chromatic fusion frequency in our experiments, the same effect would be generally observed with stimuli other than the backward background motion. We therefore examined the effect of pursuit on the detection of chromatic flicker patterns as shown in Figure 4A, B. The result (Figure 4C) indicates that chromatic fusion frequency during pursuit was similar to that during fixation. Some observers showed a decrease in performance during pursuit in comparison with during fixation, although the mean difference was not statistically significant (*p* = 0.185). This result is inconsistent with the hypothesis that the temporal resolution improvement we found is a result of the general increase in chromatic contrast sensitivity [10], [14]. On the other hand, the result is consistent with the enhanced suppression of motion blur for patterns from the stationary environment. This is because the flicker pattern of the pursuit condition (Figure 4A) was not perceived to be stationary in the environment, but to be moving together with the tracking target. Red and green appeared to alternate within the same moving bars, just as they did within the same stationary bars under the fixation condition (Figure 4B). In this case, trajectory integration should facilitate color integration rather than color segregation. In agreement with this interpretation, the chromatic fusion frequency of the flicker stimulus was ∼5 Hz lower than that of comparable motion stimulus (Figure 2B).

Furthermore, in agreement with a previous finding that the reduction of subjective motion blur during pursuit is direction selective [15], [16], we also found the temporal resolution improvement during pursuit was direction selective. In this experiment, while the observer was making a pursuit, we presented the bars moving at a display speed twice as fast as the pursuit target (Figure 4 D). On the retina, the image motion was identical to that produced by stationary bars, except that the direction was the same as, rather than opposite to, the pursuit direction (Figure 4 E). The results (Figure 4 C) indicate that chromatic fusion frequency measured under this condition (20.1 Hz±0.4) was not significantly different from that of the fixation condition (Figure 2A, 21.2 Hz±0.9, p = 0.296). Again, this finding indicates that the temporal resolution improvement during pursuit is not a result of the general increase in chromatic contrast sensitivity [10], [14], but a result of enhanced motion deblur of the stationary environment.

## Discussion

Here we demonstrate that the critical chromatic fusion frequency is elevated for retinal color changes generated by pursuit-induced background motion relative to the same changes generated by an object motion during eye fixation. This is unlikely to be a result of increased retinal misalignments of red and green bars for the pursuit condition, because we matched the range of retinal jitter between the pursuit and fixation conditions and because pursuit did not improve chromatic temporal resolution under other conditions (Figure 4).

The present finding indicates that the temporal resolution of chromatic perception is not simply determined by the temporal resolution of peripheral encoding of chromatic signals. In agreement with this conclusion, previous physiological [3], [4], psychophysical [5], [6] and imaging [7] studies have indicated that chromatic temporal modulations well above the perceptual fusion limit can reach visual cortical areas. The present study further suggests that extra-retinal neural signals related to eye movements can improve temporal resolution of color signals. This implies that cortical modulation of chromatic temporal responses are not determined within visual color processing, but controlled by a broad neural network including interactions with non-visual information.

The present finding can be considered as a novel motion deblur effect. One proposed mechanism of motion deblur is neural integration of visual signals along the trajectory of moving objects [8], [17], [18]. Our group recently reported a couple of psychophysical phenomena that demonstrate that the visual system indeed integrates color signals along the motion trajectory. One is that different colors presented at separate retinal locations, but along the same trajectory of a moving object, are perceptually mixed [13]. The other is that different colors presented at the same retinal location, but along separate motion trajectories, are perceptually segregated more than expected from retinal flicker ([11]; see also [19]). The present study further shows that the second phenomenon, motion-induced color segmentation, is enhanced by pursuit. Given that, we suspect that the role of pursuit might be to enhance trajectory integration of color signals. A possible scenario is that extra-retinal signals about pursuit enhance motion signal in the direction of the background motion during pursuit. This directional enhancement in turn facilitates color integration along that trajectory but suppresses color integration along the retinally stationary trajectory. Since the signal integration along the retinally stationary trajectory would cause motion blur under ordinary situations, suppressing it will reduce motion blur. A previous study indicates that the contrast sensitivity of luminance grating for that direction is reduced during pursuit [20]. However, our recent finding [21] indicates that the apparent strength supra-threshold motion for that direction is rather enhanced relative to the opposite direction in agreement with the scenario. We speculate that the neural mechanism responsible for the pursuit enhancement of chromatic temporal resolution is modulation of color processing areas (e.g., V4) by motion processing areas (e.g., MT and MST) [22], with eye movement signals feeding those areas [23], [24].

It is being realized that eye movements not only change gaze directions but also modulate visual perception in various ways. In comparison with the effects of saccades on visual perception [25]–[28], relatively little is known about the effect of pursuit. There are however two notable exceptions, both of which appear relevant to the present finding. One is the elevation of chromatic contrast sensitivity before and during pursuit allegedly due to an increased contrast gain of parvocellular mechanisms [10], [14]. As noted above, we think the general parvocellular gain increase is not what we revealed in the present study, since we did not find any improvement of temporal resolution when we used a pattern flickering or moving in the reversed direction on the retina. The two studies differ from each other in various aspects, however. Schütz et al. [10], [14] measured the contrast detection threshold for a horizontal chromatic line presented on the equiluminant background, while we measured the critical fusion frequency for a chromatic bar grating presented on the dark background. The stimulus of Schütz et al. [10] was designed to minimize the contribution of the luminance motion mechanism, while our stimulus was designed to maximize it. We consider that this difference is why the two studies reveal different perceptual enhancement mechanisms operating during pursuit.

One may however consider that the finding by Schütz et al. [10], [14] is related to the present findings in that both show elevations of chromatic sensitivity for stationary background—unlike our flicker control stimulus, the horizontal line flash used by Schütz et al. is apparently stationary in the environment. Although we are now considering that separate neural mechanisms are responsible for the two findings, it is also an interesting direction of future study to seek for a possibility that a common and elaborated neural mechanism may be responsible for the two findings.

The second related finding is that suppression of apparent blur of moving stimulus [12] is enhanced during pursuit [9]. This motion deblur enhancement was only observed when the untracked target moved in the opposite direction of pursuit [15], [16]. Given that the motion deblur implies a reduction in signal mixture among spatially adjacent inputs, its enhancement during pursuit is consistent with our finding of increased temporal resolution. Both indicate a function of the visual system to reduce background motion blur during pursuit. However, while Bedell and his colleague studied deblur of luminance signals, we examined deblur of color signals. Since chromatic temporal response is more sluggish than achromatic response, motion blur is a more serious problem for color than for luminance signals. The more critical difference is that while Bedell and his colleague dealt with subjective motion deblur, we showed an improvement in objective performance. There has been a debate as to whether motion deblur is accompanied by objective temporal sharpening or is just a change in subjective appearance [29], [30]. Our finding demonstrates that motion deblur during pursuit is indeed accompanied by objective temporal sharpening at least for chromatic signals. Bedell and his colleague also reported an objective performance change in the form of a 10% increase of natural temporal frequency of the temporal impulse response function [31]. However, their data only indicate a slight sensitivity decrease for lower temporal frequencies, showing no increase in sensitivity for high temporal frequencies at all. The phenomenon investigated by Bedell and his colleague looks similar to the present one in various aspects, but it remains unclear whether the two phenomena share a common mechanism. This is an issue awaiting further investigation.

In conclusion, the present study shows for the first time that the temporal resolution of color processing can be improved by a cortical process. This is a novel pursuit enhancement of visual perception. The underlying mechanism is likely to be an enhancement of trajectory integration of color signals rather than a change in gain or shape of chromatic temporal response function.

## Methods

### Observers

Observers were two of the authors (M.T. & S.N.) and three to five volunteers who were unaware of the purpose of the experiments. All have normal or corrected-to-normal vision. Informed consent was obtained after the nature and possible consequences of the studies were explained.

### Apparatus

The visual stimulus was displayed on a GDM-F520 CRT monitor (Sony), with a refresh rate of 160 Hz, driven by a VSG2/5 visual stimulus generator (Cambridge Research Systems Ltd.) installed in a Precision 350 workstation (Dell). The spatial resolution of the monitor was 800×600 pixels, with each pixel subtending 1.5 min at the viewing distance of 113 cm. The observer sat with his or her head fixed on a chin rest and viewed the display binocularly. The room had no illumination except for the stimulus presented on the CRT. A keyboard was placed in front of the observer for recording of responses. The movements of the dominant eye were monitored at 500 Hz with EyeLink II (SR research Ltd.). The analog output data by Eyelink II were recorded on disk for off-line analysis using a data acquisition system (NR-2000; Keyence) at a 1-kHz sampling rate.

### Experiment 1

The stimulus consisted of two horizontal arrays of vertical bright bars. Each array subtended 20° in width. The height of the each array, measured at the midline, was 1.0 deg above or 1.0 deg below (the trajectory of) the fixation point. Each bar subtended 6 min in width and 1.0 deg in height. The background was a dark field. Each array was a spatial alternation of two types of bar, *B_odd_* and *B_even_*. The intensity and chromaticity of the bars *B_odd_*, *B_even_* were varied by changing the intensities of red (CIE, 1931; x = 0.625, y = 0.341) and green (x = 0.290, y = 0.606) phosphors. To reduce motion ambiguity at short inter-bar intervals, we gave a luminance pedestal to either *B_odd_* or *B_even_* (11). Let us assume that (R, G)  =  (1.0, 1.0) implies an intensity pair of maximum intensity red (18.1 cd/m^2^) and equiluminant green. With no color modulation, the R-G values are *B_odd_* =  (0.25, 0.25) and *B_even_* = (0.25+0.25, 0.25+0.25) = (0.5, 0.5). This setting gives rise to an achromatic pattern made of dark and bright yellow bars. In this and the following examples, the luminance pedestal is always given to *B_odd_*. With the full chromatic modulation, the R-G values are *B_odd_* = (0.5, 0.0) and *B_even_* = (0.0+0.25, 0.5+0.25) = (0.25, 0.75), or *B_odd_* = (0.0, 0.5) and *B_even_* = (0.75, 0.25). This setting gives rise to a chromatic pattern made of red and green bars. The color without the luminance pedestal has the maximum saturation of the display, while the color with the luminance pedestal is slightly desaturated. The chromatic contrast, which we defined as |(R_1_−R_2_−G_1_+G_2_)/(R_1_+R_2_+G_1_+G_2_)|, was 67%. With the half chromatic modulation, *B_odd_* =  (0.375, 0.125) and *B_even_* =  (0.125+0.25, 0.375+0.25) = (0.375, 0.625), or *B_odd_* =  (0.125, 0.375) and *B_even_* =  (0.625, 0.375). The chromatic contrast was 33%. Given the constant speed of retinal motion of the bar arrays, the retinal red-green alternation rate depends on the inter-bar spacing. At the retinal speed of 16 deg/s, the retinal image shifts 6 min (one bar width) every 6.25 ms (one frame of the CRT monitor). The alternation rate increased from 13.3 to 26.7 Hz as the inter-bar spacing decreased from 36 to 18 min.

When a trial started, two stationary bar arrays were shown in yellow with no chromatic modulation, with an inter-bar interval chosen randomly for each trial. Under the pursuit condition, the arrays remained stationary throughout a trial. Following a key press by the observer, a bright white point moved horizontally at a constant speed of 16 deg/s, for 20 deg (1.25 s) from the left end to the right end of the CRT screen. The observer had to make a pursuit eye movement to track that point. For assistance of stable pursuit, several darker points moved along the same path together with the fixation point. When the fixation point reached the screen center area, one of the arrays briefly changed into a red-green alternating grating either at high or low chromatic contrast (Figure 1 A, B). Under the fixation condition, following a key press by the observer, the bar arrays moved horizontally for 1.25 ms at a constant speed of 16 deg/s, while the observer fixated on the central stationary point. A target color change occurred at the middle of stimulus presentation (Figure 1 C, D). Under both conditions, the target presentation was 187 ms in total, including 62.5-ms linear transition periods at the onset and offset. The observers had to make a two-alternative forced choice judgment about which stimulus was the target.

Calibration of the eye movement system was carried out at the beginning of each block. The stimulus condition (pursuit/fixation), target chromatic contrast and the direction of retinal motion were changed between blocks.

From the psychometric function obtained for each observer and condition, we estimated the critical chromatic fusion frequency from the 75% correct point of the best fitting logistic function. To minimize the difference in the magnitude of retinal jitter between the two stimulus conditions, we computed the threshold of the pursuit condition only from those trials in which retinal deviation from the expected fixation trajectory was comparable to that of the fixation condition. This was done in the following way. To quantify the magnitude of eye deviation, we calculated the RMSE (root mean square error) of horizontal eye position in the least-square fitting of the obtained eye trace to an ideal linear function, y = ax+b, where x is the horizontal eye position, a is the expected speed (0 deg/s for the fixation condition; 16 deg/s for the pursuit condition), and b is a free position offset parameter. This analysis was applied to a 300-ms interval from 56.25 ms before until 56.25 after the target presentation period. For the fixation condition, we excluded trials in which the RMSE >0.2 deg. For the pursuit condition, we excluded trials in which the RMSE exceeded the range of the RMSE shown by that observer under the fixation condition. Note that this procedure excluded trials with catch-up saccades. No valid trials contained rapid eye movements exceeding 30 deg/s. We collected data until there were at least 18 valid trials for each data point.

### Experiment 2

To make the retinal image identical between the pursuit and fixation conditions throughout a stimulus presentation, we moved the whole bar array together with bars under the fixation condition. We reduced the width of the bar array to 5 deg, and the total moving distance to 15 deg. When a chromatic target was presented, at the left and right edges of the array, flashes of some colored bars did not follow, or were followed by, flashes of different color bars on the retina. In the first experiment, this edge effect occurred in the periphery, and thus was unlikely to be critical. With a stimulus smaller than used in the second experiment, however, the edge effect might not be negligible. We therefore linearly reduced the chromatic contrast from the maximum value to zero as the spatial position changed from the center to both ends of the array.

### Experiments 3 and 4

We used a procedure identical to that for the full contrast condition of Experiment 1, except for the stimulus spatiotemporal pattern.
